# Supplementary material for: Post-acute sequelae of COVID-19 in residents in long-term care homes: Examining symptoms and recovery over time
Source: PLoS One. 2025 May 5;20(5):e0321295. doi: 10.1371/journal.pone.0321295 (PMC12052191; doi:10.1371/journal.pone.0321295)
Supplement: S1 Text — (PDF) [file pone.0321295.s001.pdf]

S1 File. Additional Information

Symptoms

List of symptoms (alphabetic) reviewed in the medical charts: abdominal pain, agitation, anxiety, appetite loss, awareness reduced, body aches, breathing shortness, cant feel body parts, chest congestion, chest pain, chills, consciousness loss, cough, dehydration, depressed mood, diarrhoea, disorientation, dizziness, fainting, fatigue, fever, flare-up, gait, hallucinations, headache, hearing loss, hoarse voice, internal bleeding, joint pain, lumpy lesions, low food intake, low fluid intake, myalgia, nasal congestion, nausea, other pain, palpitation, peripheral edema, rashes, ringing in the ears, sleep trouble, smell/taste loss, sore/throat, speak/understand loss, swallow/chew problem, thrombosis, tremors, vision problem, vomiting, bedsore pressure ulcer, ulcer from skin disease, other symptoms.

Missing Data

Information about symptoms was not available for 26 residents, and they were excluded from the relevant analyses that involved clinical symptoms (total n = 433).
During the follow-up, mortality was reported for 30 residents. Additionally, a small number of residents (n = 12) moved out during the study period. For these residents, all available information was included in the analyses prior to the death date/move out date. Therefore, total n in analyses regarding symptoms in different time periods varied slightly (n in PRE - POST2 = 433, n in POST3 = 423, n in POST4 = 414 and n in POST5 = 403), Fig 3 and 4.
In the recovery trajectories analysis, due to incomplete information about the outcome for move-out residents, their data was not included. After excluding cases with symptom-info missing and move-outs, total n for recovery trajectories analysis was 423 (T1 n = 109, T2 n = 209, and T3 n = 107), Table 1.
For residents for whom mortality was reported during the follow up period (included in numbers listed above) – most of these residents were classified in T1 trajectory, as increase in symptoms was reported in time periods after ACUTE-COVID (20 out of 30 residents, and missing symptoms n=2 in this group).

Supporting Information for Rajlic, G., Sorensen M. J., Shams B., Mardani, A., Merchant, K., & Mithani, A. “Post-acute sequelae of COVID-19 in residents in long-term care homes: Examining symptoms and recovery over time”.
