# Supplementary material for: Post-acute sequelae of COVID-19 in residents in long-term care homes: Examining symptoms and recovery over time
Source: PLoS One. 2025 May 5;20(5):e0321295. doi: 10.1371/journal.pone.0321295 (PMC12052191; doi:10.1371/journal.pone.0321295)
Supplement: S1 Fig — (PDF) [file pone.0321295.s002.pdf]

S1 Fig. Symptoms in T1 Trajectory

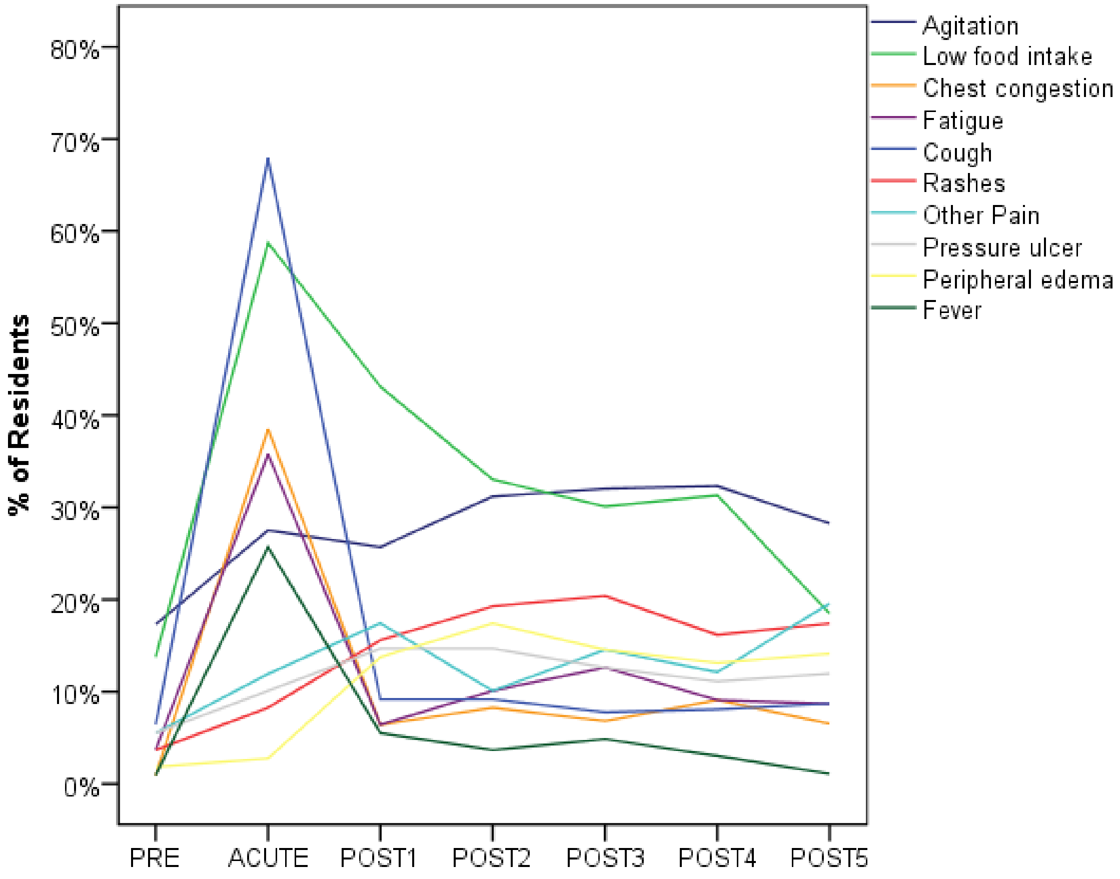

Figure S1. Most frequent symptoms in ACUTE-COVID in the group of residents in T1 trajectory (n = 109) and over all study periods.

Supporting Information for Rajlic, G., Sorensen M. J., Shams B., Mardani, A., Merchant, K., & Mithani, A. “Post-acute sequelae of COVID-19 in residents in long-term care homes: Examining symptoms and recovery over time”.
