# Supplementary material for: Post-acute sequelae of COVID-19 in residents in long-term care homes: Examining symptoms and recovery over time
Source: PLoS One. 2025 May 5;20(5):e0321295. doi: 10.1371/journal.pone.0321295 (PMC12052191; doi:10.1371/journal.pone.0321295)
Supplement: Attachment — Submitted filename: Response to Reviewers.docx [file pone.0321295.s005.docx]

Thank you for reviewing our manuscript.

Responses to Editor’s and Reviewers’ comments are in bold font in this letter (following each specific comment).

All changes made in the manuscript are indicated with track changes (that file is submitted as ‘Revised Manuscript with Track Changes’).

Revised manuscript (final version) without tracked changes is submitted as “Manuscript”.

Please let us know if anything else is needed.

Sincerely,

Gordana Rajlic, on behalf of the authors

CC: [drjahun@yahoo.co.uk](mailto:drjahun@yahoo.co.uk), [ijahun@skydalink.com](mailto:ijahun@skydalink.com)

PONE-D-25-01016

Post-acute sequelae of COVID-19 in residents in long-term care homes: Examining symptoms and recovery over time

PLOS ONE

Dear Dr. Rajlic,

Thank you for submitting your manuscript to PLOS ONE. After careful consideration, we feel that it has merit but does not fully meet PLOS ONE’s publication criteria as it currently stands. Therefore, we invite you to submit a revised version of the manuscript that addresses the points raised during the review process.

==============================
**ACADEMIC EDITOR:**

The manuscript contributes to the topic about post COVID-19 condition among elderly in LTC homes. This is critical area and very relevant toward ensuring quality care among this population not only for COVID-19 but for other flu-related conditions. The quality of the study is also near satisfactory. Please respond to the following comments in addition to the comments raised by the 2 reviewers below:

1. This study involves human subjects and therefore the ethical considerations should be clearly presented. Please create a subsection "Ethical Considerations" and consolidate all ethical decisions under this subsection.

2. Ensure that the manuscript is presented in line with plosone guidelines [Submission Guidelines | PLOS ONE](https://can01.safelinks.protection.outlook.com/?url=https%3A%2F%2Ftrack.editorialmanager.com%2FCL0%2Fhttps%3A%252F%252Fjournals.plos.org%252Fplosone%252Fs%252Fsubmission-guidelines%2F1%2F010f0194f57656ab-f4d9d3f2-20fe-49b9-aa91-ccca90a5d6b2-000000%2F9H8zlVE_HLVDTNPYk-H04CKRZwcnGOFX_0q5YH5O7Pw%3D197&data=05%7C02%7Cgordana.rajlic%40fraserhealth.ca%7C902b566198bd451690bf08dd4aaa57cd%7C31f660a5192a4db392baca424f1b259e%7C0%7C0%7C638748818344688988%7CUnknown%7CTWFpbGZsb3d8eyJFbXB0eU1hcGkiOnRydWUsIlYiOiIwLjAuMDAwMCIsIlAiOiJXaW4zMiIsIkFOIjoiTWFpbCIsIldUIjoyfQ%3D%3D%7C0%7C%7C%7C&sdata=xuecaP6bM2vWn%2F79DpxNFcYvnh%2B%2FzSHtlrTjkcQ7b14%3D&reserved=0)

==============================

Please submit your revised manuscript by  Mar 28 2025 11:59PM. If you will need more time than this to complete your revisions, please reply to this message or contact the journal office at [plosone@plos.org](mailto:plosone@plos.org). When you're ready to submit your revision, log on to <https://www.editorialmanager.com/pone/> and select the 'Submissions Needing Revision' folder to locate your manuscript file.

Please include the following items when submitting your revised manuscript:

- A rebuttal letter that responds to each point raised by the academic editor and reviewer(s). You should upload this letter as a separate file labeled 'Response to Reviewers'.
- A marked-up copy of your manuscript that highlights changes made to the original version. You should upload this as a separate file labeled 'Revised Manuscript with Track Changes'.
- An unmarked version of your revised paper without tracked changes. You should upload this as a separate file labeled 'Manuscript'.

If you would like to make changes to your financial disclosure, please include your updated statement in your cover letter. Guidelines for resubmitting your figure files are available below the reviewer comments at the end of this letter.

We look forward to receiving your revised manuscript.

Kind regards,

Ibrahim Jahun, MD, MSC, PhD
Academic Editor
PLOS ONE

Journal Requirements:

When submitting your revision, we need you to address these additional requirements.

1. Please ensure that your manuscript meets PLOS ONE's style requirements, including those for file naming. The PLOS ONE style templates can be found at 
<https://journals.plos.org/plosone/s/file?id=wjVg/PLOSOne_formatting_sample_main_body.pdf> and 
<https://journals.plos.org/plosone/s/file?id=ba62/PLOSOne_formatting_sample_title_authors_affiliations.pdf>

**We reviewed the guidelines again and made some corrections in compliance with the PLOS ONE’s style requirements (changes tracked in the manuscript).**

2. Thank you for stating in your Funding Statement: 
“The study was supported by funding from the COVID‐19 Pandemic Response and Impact Grant (Co‐RIG) Program of the Foundation for Advancing Family Medicine and the Canadian Medical Association Foundation, Grant/Award Number: FAFM-2021-0094-EN_Mithani. (A.M. and J.S.)
<https://fafm.cfpc.ca/corig/>
The funders had no role in study design, data collection and analysis, decision to publish, or preparation of the manuscript.”

Please provide an amended statement that declares *all* the funding or sources of support (whether external or internal to your organization) received during this study, as detailed online in our guide for authors at <http://journals.plos.org/plosone/s/submit-now>. Please also include the statement “There was no additional external funding received for this study.” in your updated Funding Statement. 
Please include your amended Funding Statement within your cover letter. We will change the online submission form on your behalf.

**We clarified that this reported funding was all funding/support received for the study. There was no additional external or internal funding received for this study.**

**Amended Funding Statement:**

**“The study received funding from the COVID‐19 Pandemic Response and Impact Grant (Co‐RIG) Program of the Foundation for Advancing Family Medicine and the Canadian Medical Association Foundation, Grant/Award Number: FAFM-2021-0094-EN_Mithani. (A.M. and J.S.)** [**https://fafm.cfpc.ca/corig/**](https://fafm.cfpc.ca/corig/) **There was no additional internal or external funding received for this study. The funders had no role in study design, data collection and analysis, decision to publish, or preparation of the manuscript.”**

3. We note that you have indicated that there are restrictions to data sharing for this study. For studies involving human research participant data or other sensitive data, we encourage authors to share de-identified or anonymized data. However, when data cannot be publicly shared for ethical reasons, we allow authors to make their data sets available upon request. For information on unacceptable data access restrictions, please see <http://journals.plos.org/plosone/s/data-availability#loc-unacceptable-data-access-restrictions>.

Before we proceed with your manuscript, please address the following prompts:

a) If there are ethical or legal restrictions on sharing a de-identified data set, please explain them in detail (e.g., data contain potentially identifying or sensitive patient information, data are owned by a third-party organization, etc.) and who has imposed them (e.g., a Research Ethics Committee or Institutional Review Board, etc.). Please also provide contact information for a data access committee, ethics committee, or other institutional body to which data requests may be sent.

b) If there are no restrictions, please upload the minimal anonymized data set necessary to replicate your study findings to a stable, public repository and provide us with the relevant URLs, DOIs, or accession numbers. Please see <http://www.bmj.com/content/340/bmj.c181.long> for guidelines on how to de-identify and prepare clinical data for publication. For a list of recommended repositories, please see <https://journals.plos.org/plosone/s/recommended-repositories>. You also have the option of uploading the data as Supporting Information files, but we would recommend depositing data directly to a data repository if possible.

Please update your Data Availability statement in the submission form accordingly.

**Updated Data Availability statement:**

**Data used in the study was collected by Fraser Health for health care purposes and are restricted by the Freedom of Information and Protection of Privacy Act (FIPPA) regulations. Data cannot be shared publicly because there are ethical and privacy restrictions on sharing a de-identified data set (i.e. the data contains potentially identifying and sensitive information). Researchers who meet the criteria for access to confidential data are encouraged to contact the Department of Evaluation and Research Services (research.approvals@fraserhealth.ca) to discuss how their data request can be facilitated.**

4. In the online submission form, you indicated that [The data that support the findings of this study are available upon request from the corresponding author and with appropriate Fraser Health Authority institutional and British Columbia Harmonized Ethics Review approvals for secondary use of data. Medical records data are not publicly available due to institutional regulations and privacy or ethical restrictions.]. 
All PLOS journals now require all data underlying the findings described in their manuscript to be freely available to other researchers, either 1. In a public repository, 2. Within the manuscript itself, or 3. Uploaded as supplementary information.
This policy applies to all data except where public deposition would breach compliance with the protocol approved by your research ethics board. If your data cannot be made publicly available for ethical or legal reasons (e.g., public availability would compromise patient privacy), please explain your reasons on resubmission and your exemption request will be escalated for approval.

**Our data can not be made publicly available for both ethical and legal reasons.** **These are secondary data (medial records data) released to us from Fraser Health Authority only for the purpose of conducting research. Additional approval is needed for any other uses of data. The protocol approved by the research ethics board did not include data sharing, and additional approval from the ethics is also required, upon review for privacy and ethical considerations for data sharing.**

**To respond to your additional inquiry, we contacted the regulating bodies and in consultation with them, we are providing an updated Data availability statement (the statement is more specific and answers your questions). If any additional information is needed, please let us know.**

**Updated Data Availability statement:**

**Data used in the study was collected by Fraser Health for health care purposes and are restricted by the Freedom of Information and Protection of Privacy Act (FIPPA) regulations. Data cannot be shared publicly because there are ethical and privacy restrictions on sharing a de-identified data set (i.e. the data contains potentially identifying and sensitive information). Researchers who meet the criteria for access to confidential data are encouraged to contact the Department of Evaluation and Research Services (research.approvals@fraserhealth.ca) to discuss how their data request can be facilitated.**

5. Please review your reference list to ensure that it is complete and correct. If you have cited papers that have been retracted, please include the rationale for doing so in the manuscript text or remove these references and replace them with relevant current references. Any changes to the reference list should be mentioned in the rebuttal letter that accompanies your revised manuscript. If you need to cite a retracted article, indicate the article’s retracted status in the References list and also include a citation and full reference for the retraction notice.

**We checked our reference list. We did not cite retracted papers.**

Reviewers' comments:

Reviewer's Responses to Questions

Comments to the Author

1. Is the manuscript technically sound, and do the data support the conclusions?

The manuscript must describe a technically sound piece of scientific research with data that supports the conclusions. Experiments must have been conducted rigorously, with appropriate controls, replication, and sample sizes. The conclusions must be drawn appropriately based on the data presented.

Reviewer #1: Yes

Reviewer #2: Yes

2. Has the statistical analysis been performed appropriately and rigorously?

Reviewer #1: Yes

Reviewer #2: I Don't Know

3. Have the authors made all data underlying the findings in their manuscript fully available?

The [PLOS Data policy](https://can01.safelinks.protection.outlook.com/?url=http%3A%2F%2Ftrack.editorialmanager.com%2FCL0%2Fhttp%3A%252F%252Fwww.plosone.org%252Fstatic%252Fpolicies.action%2523sharing%2F1%2F010f0194f57656ab-f4d9d3f2-20fe-49b9-aa91-ccca90a5d6b2-000000%2F5OZVfG8Ga1eKHd445wh9M0Hadv3hzSoWqGXdfh2yq10%3D197&data=05%7C02%7Cgordana.rajlic%40fraserhealth.ca%7C902b566198bd451690bf08dd4aaa57cd%7C31f660a5192a4db392baca424f1b259e%7C0%7C0%7C638748818344739656%7CUnknown%7CTWFpbGZsb3d8eyJFbXB0eU1hcGkiOnRydWUsIlYiOiIwLjAuMDAwMCIsIlAiOiJXaW4zMiIsIkFOIjoiTWFpbCIsIldUIjoyfQ%3D%3D%7C0%7C%7C%7C&sdata=0%2FxX3HSS526jWN2c8qSAMEBglmBa4Liv3UP5O7PKw8k%3D&reserved=0) requires authors to make all data underlying the findings described in their manuscript fully available without restriction, with rare exception (please refer to the Data Availability Statement in the manuscript PDF file). The data should be provided as part of the manuscript or its supporting information, or deposited to a public repository. For example, in addition to summary statistics, the data points behind means, medians and variance measures should be available. If there are restrictions on publicly sharing data—e.g. participant privacy or use of data from a third party—those must be specified.

Reviewer #1: Yes

Reviewer #2: Yes

4. Is the manuscript presented in an intelligible fashion and written in standard English?

PLOS ONE does not copyedit accepted manuscripts, so the language in submitted articles must be clear, correct, and unambiguous. Any typographical or grammatical errors should be corrected at revision, so please note any specific errors here.

Reviewer #1: Yes

Reviewer #2: Yes

5. Review Comments to the Author

Please use the space provided to explain your answers to the questions above. You may also include additional comments for the author, including concerns about dual publication, research ethics, or publication ethics. (Please upload your review as an attachment if it exceeds 20,000 characters)

Reviewer #1: The manuscript addresses an important topic which was approached using an appropriate study design, and your results justify the conclusions stated, and the manuscript is well written.

I do have some questions and feedback:

pg5 ln100: According to your methods you indicate collecting information for 5 periods of 4weeks (=20 weeks) after infection. Please clarify.

**On pg5 ln100, we stated ‘…to describe the symptoms in COVID-19 survivors over 24 weeks after infection’. We changed to ‘…over 24 weeks after contracting infection’. Thank you for pointing to this, it needed to be specified here. These 24 weeks could be further broken down to a 4-week acute-COVID and 20 weeks after the acute phase (post-acute time consisting of 5 POST periods of 4 weeks = 20 weeks). This was explained later in the text (Procedure) and here, the purpose was just to make a general statement referring to the time after contracting infection.**

**The total time, including PRE-COVID, ACUTE-COVID, and 5 post-acute POST periods, was 28 weeks. This was depicted visually in Fig 1, and we added reference to Fig 1 to several paragraphs, as visual presentation is helpful to distinguish these time periods.**

pg5 ln107: Please specify the month/year due to discrepancies in the start of the epidemic

**We added specific dates to the manuscript: from March 01, 2020 to December 31, 2021.**

pg5 ln108-109: Why did you pick this criteria given your follow-up period was 24 weeks

**This was our study inclusion (eligibility) criteria – we wanted to include residents who survived the acute disease (‘COVID-19 survivors’), and to examine symptoms in this group of residents only. It was deemed that 2-month period was sufficient in determining that the resident was beyond the most critical phase of the disease. For these residents (who met our eligibility criteria) we then collected information over 28 weeks time, in the defined study periods (i.e., pre-COVID, acute-COVID, post-COVID time). We expanded this section a bit to provide more detail on our inclusion criterion.**

**Previously, we conducted another study focusing on mortality after contracting COVID-19 (during the acute phase and over a longer period after) and we provided evidence in this respect. This current study was intended to focus on COVID-19 survivors only (and their symptoms pre, during, and after COVID), that is why we had such study eligibility criteria. If in this group mortality occurred, it was reported (in Missing data), and this mortality was not part of our study inclusion-exclusion criteria.**

pg6 ln112-113: I am not sure what the differences are between assisted living and LTC in BS but in Alberta there are considerable differences and providing different levels of care. Therefore, this last part of the sentence is confusing and contradictory.

**Wording we used here was confusing - we reworded so that it is clearer that we included LTC homes only.**

*“The LTC homes participating in the study are affiliated with Fraser Health Authority (FHA), and only LTC homes were included (i.e., homes providing 24-hour professional service to meet residents’ complex care needs) while Assisted Living facilities were not included in the study.”*

pg7 ln138-140: I am confused about this criteria - if you are looking at information about pre-existing symptoms, why are you collecting symptoms from the date of COVID-19 symptoms onset, as any symptoms collected then could be attributed to COVID? Or perhaps you meant that symptoms were collected BEFORE the date of COVID symptoms were reported? Please explain

**Yes, symptoms were collected BEFORE the day any COVID-19 symptoms were reported (if COVID-19 symptoms were reported before positive COVID-19 test date). The current wording is:**

*“For the PRE-COVID period, information about symptoms (i.e., COVID-19 unrelated symptoms) was collected before the date of positive COVID-19 test or the date that onset of COVID-19 symptoms was reported, whichever of was earlier, as noted in the chart”.*

pg16 ln319: According to your time frame, you examined symptoms before, during covid and then 5 months after. Please correct.

**Yes, that is correct. We added ‘see Figure 1’ at the end of this sentence. In Figure 1, time periods are visually presented, therefore referring to the figure should be helpful. We also added ‘contracting’ into that sentence:**

*“We examined symptoms over six months after contracting COVID-19 infection and in a period before infection, while focusing on short time increments (i.e., a 4-week PRE-COVID period, a 4-week ACUTE-COVID period, and five 4-week periods subsequent to ACUTE-COVID, see Fig 1).”*

pg16 ln333-334: But you only measured upto 5 months after covid?

**We reworded slightly, the specific time reference was removed here – it was not necessary, as specifics of the time frame were explained in many places throughout the manuscript.**

*“The results revealed that even though many COVID-19 survivors (about a half of them) returned to their PRE-COVID symptom level after ACUTE-COVID, a quarter of the residents did not return to their baseline symptom level over the length of the follow-up period”.*

**To Reviewer 1: Thank you very much for thoroughly reading through the manuscripts and for your comments. They were helpful to us, to see where clarification was needed and to improve the manuscript.**

Reviewer #2: This study contributes evidence about symptoms over time in 459 residents in nine Canadian LTC homes and has certain study significance. However, the following concerns should be addressed:

1、This retrospective study may introduce potential biases into the collected data.

**The current study was designed as retrospective examination of the information in residents’ medical charts. While prospective design is preferred, this was not realistic for us in the context of the current project. Rather, we decided to use information that was already there, in residents’ charts, collected from the onset of the pandemic. Information from regular medical examinations is valuable, and we utilized it to learn more about recovery after surviving the acute phase of COVID-19.**

**We were aware and observant of the risk for bias in the given research design, and we tried to minimize it by following our research protocol and procedure. In limitations, in Discussion section, we stated:**

“We utilized a retrospective longitudinal chart review study design, and prospective design with standardized recording of symptoms could provide further evidence about PCC outcomes. Additionally, for a full picture of PCC, information directly reported by the residents should be examined along with chart review”

**We hope that more information and in particular prospective research will be available about this topic in near future.**

2、This study indicates that the author gathered data on clinical symptoms documented in hospital medical records. Would you like to know whether there are corresponding standards for reference for these symptom information collected in the paper? Such as cognition, anxiety, etc. Are these symptoms assessed on a scale or?

**As you noted, we collected information about clinical symptoms as documents in residents’ medical records. Detailed information, such as are scale-assessed symptoms is not typically present in medical records. Therefore, we collected data about the presence/absence of the symptoms, as it was indicated in the charts. A composite score (the number of different symptoms) was the main measure that we used in the study.**

**It should be noted that symptoms were recorded by LTC professionals, according to their professional standards. For the current retrospective examination, we just collected this information, without any further interpretation/modification.**

**Upon reviewer’s comment, we added to our limitations paragraph (Discussion), and stated:**

*“We utilized a retrospective longitudinal chart review study design, and prospective design with standardized recording of symptoms, and on a finer scale, could provide further evidence about PCC outcomes.”*

**To conclude, we agree that future research that utilizes a finer scale as opposed to dichotomous categorization will provide more information about this important topic.**

**Thank you for your thoughtful comments.**

Thank you!

6. PLOS authors have the option to publish the peer review history of their article ([what does this mean?](https://can01.safelinks.protection.outlook.com/?url=https%3A%2F%2Ftrack.editorialmanager.com%2FCL0%2Fhttps%3A%252F%252Fjournals.plos.org%252Fplosone%252Fs%252Feditorial-and-peer-review-process%2523loc-peer-review-history%2F1%2F010f0194f57656ab-f4d9d3f2-20fe-49b9-aa91-ccca90a5d6b2-000000%2FEO5iHxmDW55fmeUpaWuYZrpiJxxgq4DNkCs7y92hBh8%3D197&data=05%7C02%7Cgordana.rajlic%40fraserhealth.ca%7C902b566198bd451690bf08dd4aaa57cd%7C31f660a5192a4db392baca424f1b259e%7C0%7C0%7C638748818344778967%7CUnknown%7CTWFpbGZsb3d8eyJFbXB0eU1hcGkiOnRydWUsIlYiOiIwLjAuMDAwMCIsIlAiOiJXaW4zMiIsIkFOIjoiTWFpbCIsIldUIjoyfQ%3D%3D%7C0%7C%7C%7C&sdata=mkVd%2Fw%2BZTePSoBmd9jJ34RxV4uFaktMjSomGIaT4Tfo%3D&reserved=0)). If published, this will include your full peer review and any attached files.


If you choose “no”, your identity will remain anonymous but your review may still be made public.


**Do you want your identity to be public for this peer review?** For information about this choice, including consent withdrawal, please see our [Privacy Policy](https://can01.safelinks.protection.outlook.com/?url=https%3A%2F%2Ftrack.editorialmanager.com%2FCL0%2Fhttps%3A%252F%252Fwww.plos.org%252Fprivacy-policy%2F1%2F010f0194f57656ab-f4d9d3f2-20fe-49b9-aa91-ccca90a5d6b2-000000%2FafLzGE1_8-NsVUjujicHfOSXbuE4ynvmsHF5YBQW5f4%3D197&data=05%7C02%7Cgordana.rajlic%40fraserhealth.ca%7C902b566198bd451690bf08dd4aaa57cd%7C31f660a5192a4db392baca424f1b259e%7C0%7C0%7C638748818344815346%7CUnknown%7CTWFpbGZsb3d8eyJFbXB0eU1hcGkiOnRydWUsIlYiOiIwLjAuMDAwMCIsIlAiOiJXaW4zMiIsIkFOIjoiTWFpbCIsIldUIjoyfQ%3D%3D%7C0%7C%7C%7C&sdata=ymxeD6sk3fOptvw9CfHo0TrnOEXDpmCCv8Xbj31vwkk%3D&reserved=0).

Reviewer #1: No

Reviewer #2: No

[NOTE: If reviewer comments were submitted as an attachment file, they will be attached to this email and accessible via the submission site. Please log into your account, locate the manuscript record, and check for the action link "View Attachments". If this link does not appear, there are no attachment files.]

While revising your submission, please upload your figure files to the Preflight Analysis and Conversion Engine (PACE) digital diagnostic tool, [https://pacev2.apexcovantage.com/](https://can01.safelinks.protection.outlook.com/?url=https%3A%2F%2Ftrack.editorialmanager.com%2FCL0%2Fhttps%3A%252F%252Fpacev2.apexcovantage.com%252F%2F1%2F010f0194f57656ab-f4d9d3f2-20fe-49b9-aa91-ccca90a5d6b2-000000%2FS8NrYDU2ZXEJkJVibnueC1Zp47IEMwTxDT-wHJPjYao%3D197&data=05%7C02%7Cgordana.rajlic%40fraserhealth.ca%7C902b566198bd451690bf08dd4aaa57cd%7C31f660a5192a4db392baca424f1b259e%7C0%7C0%7C638748818344848032%7CUnknown%7CTWFpbGZsb3d8eyJFbXB0eU1hcGkiOnRydWUsIlYiOiIwLjAuMDAwMCIsIlAiOiJXaW4zMiIsIkFOIjoiTWFpbCIsIldUIjoyfQ%3D%3D%7C0%7C%7C%7C&sdata=pGQpoP6ad3DbQJNKqEGfotlg43t4wDR0JsElmMPL6Us%3D&reserved=0). PACE helps ensure that figures meet PLOS requirements. To use PACE, you must first register as a user. Registration is free. Then, login and navigate to the UPLOAD tab, where you will find detailed instructions on how to use the tool. If you encounter any issues or have any questions when using PACE, please email PLOS at [figures@plos.org](mailto:figures@plos.org). Please note that Supporting Information files do not need this step.

**We completed this**.

Thank you.

*In compliance with data protection regulations, you may request that we remove your personal registration details at any time.* [*(Remove my information/details)*](https://can01.safelinks.protection.outlook.com/?url=https%3A%2F%2Ftrack.editorialmanager.com%2FCL0%2Fhttps%3A%252F%252Fwww.editorialmanager.com%252Fpone%252Flogin.asp%253Fa%3Dr%2F1%2F010f0194f57656ab-f4d9d3f2-20fe-49b9-aa91-ccca90a5d6b2-000000%2FqbKEY0LHCygvx3nvV1O7L1YMGY0IdRJ1UZdIv-SWBis%3D197&data=05%7C02%7Cgordana.rajlic%40fraserhealth.ca%7C902b566198bd451690bf08dd4aaa57cd%7C31f660a5192a4db392baca424f1b259e%7C0%7C0%7C638748818344875296%7CUnknown%7CTWFpbGZsb3d8eyJFbXB0eU1hcGkiOnRydWUsIlYiOiIwLjAuMDAwMCIsIlAiOiJXaW4zMiIsIkFOIjoiTWFpbCIsIldUIjoyfQ%3D%3D%7C0%7C%7C%7C&sdata=ZE3994%2B4KQcr20%2BmKRJks7WkC65dwGW8xg4F8juza38%3D&reserved=0)*. Please contact the publication office if you have any questions.*
